# Seroprevalence of Bactericidal, Specific IgG Antibodies and Incidence of Meningitis Due to Group A *Neisseria meningitidis* by Age in Burkina Faso 2008

**DOI:** 10.1371/journal.pone.0055486

**Published:** 2013-02-14

**Authors:** Caroline L. Trotter, Seydou Yaro, Berthe-Marie Njanpop-Lafourcade, Aly Drabo, Sita S. Kroman, Regina S. Idohou, Oumarou Sanou, Leah Bowen, Helen Findlow, Serge Diagbouga, Bradford D. Gessner, Ray Borrow, Judith E. Mueller

**Affiliations:** 1 School of Social and Community Medicine, University of Bristol, Bristol, United Kingdom; 2 Centre Muraz, Bobo-Dioulasso, Burkina Faso; 3 Agence de Médecine Préventive, Paris, France; 4 Health Protection Agency Vaccine Evaluation Unit, Manchester Royal Infirmary, Manchester, United Kingdom; 5 Institut de Recherche en Sciences de la Santé, Ouagadougou, Burkina Faso; 6 Inflammation Sciences Research Group, School of Translational Medicine, University of Manchester, Manchester, United Kingdom; 7 Department of Epidemiology and Biostatistics, Ecole des Hautes Etudes en Santé Publique, Paris, France; National Taiwan University Hospital, Taiwan

## Abstract

**Background:**

We investigated serological correlates of protection against *Neisseria meningitidis* serogroup A (NmA) in Burkina Faso before the introduction of NmA conjugate vaccine.

**Methodology/Principal Findings:**

We collected blood from a representative sample (N = 1022) of Bobo-Dioulasso residents. Sera were evaluated for serum bactericidal antibody (SBA) activity against NmA strains of immunotype L11 (F8238) and L10 (3125) and NmA-specific IgG. Seroprevalence was compared to the age-specific NmA meningitis incidence in Bobo-Dioulasso during March 2007–February 2008. Meningococcal carriage was evaluated in a subset (N = 538). Geometric mean titres (GMT)/concentrations (GMC) of SBA and NmA-specific IgG increased with age, peaking around age 20 years. Overall, 70% of our sample had NmA-specific IgG ≥2 ug/mL. Meningitis incidence was highest in those aged <6 months and 5–19 years. No NmA carriers were found. Compared to the reference strain SBA, GMTs were higher against a locally isolated strain and around 40-fold lower against Dutch strain 3125.

**Conclusions/Significance:**

This study provides estimates of natural immunity to NmA, according to a variety of antibody measures, which will be helpful in ascertaining antibody persistence after MenAfriVac™ introduction. Age-specific seroprevalence of reference strain SBA titres most likely reflects exposure to meningococci and consecutive reactive immunity. We could not define any serological correlate of protection.

## Introduction

Burkina Faso lies in the African meningitis belt, characterized by high incidence of meningococcal disease during the dry season and by the periodic occurrence of epidemics at a local or regional level [1]; [2]. Most epidemics have been caused by *Neisseria meningitidis* serogroup A (NmA), although serogroups C [3], W135 [4] and X[5]; [6] have also been implicated. Reactive immunization campaigns with meningococcal polysaccharide vaccines when implemented early during an epidemic appear to reduce its duration [7]. However, preventive rather than reactive vaccination is likely to be much more effective and to this end, a monovalent NmA conjugate vaccine (MenAfriVac™) has been developed with the aim of preventing and ultimately eliminating NmA epidemics [8]. This vaccine was first introduced in Burkina Faso and parts of Mali and Niger during late 2010 in mass campaigns targeting individuals aged 1- to 29-years [9].

As clinical effectiveness is difficult to establish, immunogenicity studies have made an important contribution to the evaluation of meningococcal vaccines. Studies on immunity to the meningococcus conducted by Goldschneider in the 1960s defined correlates of protection for serogroup C disease and demonstrated that the age-related incidence of disease in the USA was inversely correlated with the presence of serum bactericidal antibody (SBA) activity against all studied serogroups [10]. These analyses have been updated recently for serogroups B, C, W and Y in the UK [11]–[13], but have never been performed in the African meningitis belt. While SBA is thought to be the best correlate of protection for meningococcal disease [14], the only current established correlate of protection for NmA is based on serogroup- specific IgG antibodies (≥2 µg/mL) following receipt of NmA polysaccharide vaccine [15]; [16].

In this context, the objectives of our study were several. First, we sought to measure the natural seroprevalence of IgG and SBA antibodies against NmA in the meningitis belt. Second, we evaluated whether a relationship existed between the age distributions of meningitis incidence and seroprevalence [6]; [17]. Third, since population immunity may be affected by the extent of local NmA circulation, we also estimated the prevalence of meningococcal carriage in this population.

## Methods

### Ethics Statement

The study received ethical approval from the ethics committees of Centre Muraz, the Ministry of Health of Burkina Faso, and the Faculty of Medicine & Dentistry, University of Bristol.

### Recruitment and Data/Sample Collection

We included a representative sample of residents of urban Bobo-Dioulasso, aged 1 month to 59 years. With around 600,000 inhabitants, Bobo-Dioulasso is the second largest city in Burkina Faso. Preventive mass immunization campaigns with serogroup A/C polysaccharide vaccine were conducted in 2002 and after the end of the present study in March 2008. Potential participants were identified using a three-stage cluster sampling method in administrative sectors, cross-roads and compounds, as described previously [18]. If an individual refused participation, or if no person of the required age group resided in that compound, the closest neighbouring compound was contacted until up to 5 individuals from specified age-groups had been recruited for each starting point. Individuals were excluded from the study if they had a serious illness or bleeding disorder.

On recruitment during household visits, participants or their guardians (for minors <18 years) provided written, informed consent, and completed a questionnaire to assess medical history, vaccination history, and demographic and life-style information. Study visits took place at Centre Muraz between February 28 and March 7, 2008. After administration of a second questionnaire, a blood sample of 2–5 mL was taken from each participant. Pharyngeal swabs were taken from participants aged 1 month to 59 years attending the study centre after February 29, while inclusions progressed in all administrative sectors during the study along the list of cross-roads. Weight and height measurements were obtained from children <10 years of age in a standardized way using available clinical scales. Malnutrition was defined for children <121 cm tall as weight for height below the third percentile according to WHO child growth standards [19]. Children <18 years old were asked to provide oral assent before sample collection.

### Laboratory Methods and Analyses

Blood samples were centrifuged at the laboratory within 2 hours of collection and the sera were frozen at −70°C or below until analysis at the Health Protection Agency Vaccine Evaluation Unit, Manchester, UK. SBA activity was measured using baby rabbit serum (Pel-Freeze) as the exogenous source of complement [20]. SBA was assessed against the NmA reference strain (strain F8238, phenotype A:4/21:P1.20,9, clonal complex [cc] 5 ) [20] and two additional strains: M08.0240451 (phenotype A:4/21:P1.20,9, cc5; “the Burkina strain”), isolated from a meningitis case in Bobo-Dioulasso during the study period and strain 3125 (phenotype A:NT:P1.5,2, ST-1 clonal complex; “the Dutch strain”), originally isolated by the Netherlands Reference Laboratory for Bacterial Meningitis. The Dutch strain is immunotype L10, whereas the reference strain is immunotype L11 (the immunotype of the Burkina strain is currently unknown). In some cases, the available serum was insufficient to allow additional SBA testing (table 1). SBA titers were expressed as the reciprocal of the final serum dilution giving 50% killing at 60 minutes. For computational purposes, titers <4 were assigned a value of 2. Measures of IgG serum antibody concentrations to serogroups A, C, W135 and Y were obtained using a multiplex bead assay [21]. The lower limit of detection was 0.08 µg/mL for serogroup A; for any values recorded below this threshold, a value equal to 0.04 µg/mL (half the lower limit) was assigned for computational purposes.

**Table 1:** Geometric mean titres (GMT) and geometric mean concentrations (GMC) of bactericidal and IgG antibody against meningococcal serogroup A.

	Serum Bactericidal Antibody GMT (95% CI)			Anti-A IgG GMC (95% CI)
Age group (sera tested*)	Reference strain F828	Burkina strain	Dutch strain 3125	
<6 months (N = 53)	2.4 (1.8, 3.2)	2.5 (1.8, 3.4)	2.1 (2.0, 2.3)	1.2 (0.9, 1.5)
6–11 months (N = 52)	2.9 (2.0, 4.3)	2.5 (1.8, 3.5)	2.0 (2.0, 2.0)	1.6 (1.4, 1.8)
1–4 years (N = 109)	60.4 (36.6, 99.7)	74.1 (40.3, 136.4)	2.4 (2.0, 2.9)	1.2 (1.0, 1.4)
5–9 years (N = 108)	205.8 (99.2, 427.0)	484.9 (244.9, 960.1)	4.3 (2.6, 7.2)	2.2 (1.8. 2.6)
10–14 years (N = 104)	234.8 (118.9, 463.4)	461.0 (285.4, 744.4)	5.3 (2.8, 9.9)	4.1 (3.2, 5.4)
15–19 years (N = 104)	270.0 (166.1, 438.9)	620.5 (445.1, 865.0)	11.1 (6.6, 18.6)	6.9 (5.5, 8.7)
20–24 years (N = 105)	445.7 (298.2, 666.3)	708.9 (438.6, 1145.8)	12.5 (8.7, 17.9)	8.7 (7.1, 10.8)
25–29 years (N = 105)	230.3 (147.3. 360.2)	347.6 (209.3, 577.3)	9.6 (4.9, 18.5)	10.0 (6.9, 14.4)
30–39 years (N = 99)	123.6 (61.4, 248.9)	269.3 (146.6, 494.4)	9.9 (5.3, 18.6)	10.9 (6.9, 17.1)
40+years (N = 102)	103.7 (61.1, 176.0)	144.0 (92.0, 225.5)	7.2 (4.4, 11.8)	8.5 (7.0, 10.3)

Residents of Bobo-Dioulasso, Burkina Faso (N = 941) aged 1 month to 49 years, February–March 2008.

* For analyses with Burkina and Dutch strain, fewer sera were tested in some age groups, due to limited sample volume.

Pharyngeal swabs behind the uvula were obtained via the mouth using cotton-tipped sterile swabs, which were placed in a protective capsule and transported to the laboratory for inoculation onto selective medium within five minutes of collection. Plates were stored in a 5% CO2 atmosphere at room temperature until incubation at 37°C. Meningococci were isolated and identified using established bacteriologic methods and PCR at the Centre Muraz, Burkina Faso [18]. Isolates were sent to the Institut de Medicine Tropicale du Service de Santé des Armées (IMTSSA), Marseilles, France for sero(sub)typing by standard methods, and genotyped by pulsed field gel electrophoresis and multi-locus sequence typing as previously described [22]; [23].

### Statistical Analyses

**Figure 1:**
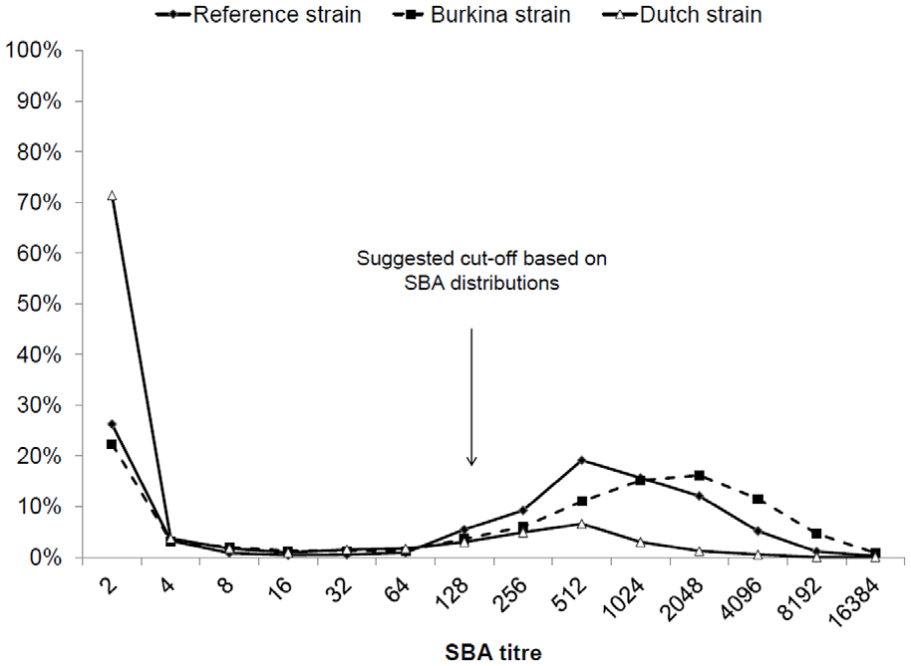
Distribution of serum bactericidal antibody (SBA) titres against three meningococcal serogroup A assay strains. Residents of Bobo-Dioulasso, Burkina Faso (N = 941) aged 1 month to 49 years, February–March 2008.

All analyses were conducted in Stata (v11.0) accounting for design effect induced by cluster sampling. We excluded participants self-reporting meningococcal vaccination during 2007/8. Age-specific geometric mean titers (GMT) for SBA measurements and geometric mean concentrations (GMC) for NmA-specific IgG were calculated and examined graphically using standard methods. To evaluate differences, t-tests were performed on log-transformed variables. We examined three different SBA titer cut-offs as hypothetical correlates of protection: 8, based on the literature and previous seroprevalence studies in this population [18]; 128, based on the distribution of titers found among participants of the present study (Figure 1); and 1024, which has been suggested as a cut-off for epidemic serogroup A meningitis [24]. NmA-specific IgG concentrations ≥2 µg/mL were assumed putatively protective [15]; [16]. The association between the log-transformed serogroup A SBA titers and IgG antibody concentrations were examined using Pearson’s correlation coefficients and linear regression. Factors associated with SBA titers and IgG concentrations above specified cut-offs were examined using multivariable logistic regression.

### Incidence Data

Meningitis incidence data were obtained from a concomitant exhaustive surveillance study in the urban area of Bobo-Dioulasso, as previously reported [6]; [17]. Briefly, all suspected meningitis cases identified in any of the hospitals or health centres in the surveillance area were enrolled, lumbar puncture performed and cerebrospinal fluid evaluated by culture, latex and PCR. The surveyed population was approximately 600,000 inhabitants in the urban parts of sanitary districts Do and Dafra during March 2007 through February 2008. We chose this period to have an entire year comprising both rainy (endemic) and dry (hyperendemic) seasons and because a mass campaign with Nm serogroup A/C polysaccharide vaccine was implemented in the study population in mid-March 2008, after the seroprevalence study. Annual and monthly age-specific incidence rates for laboratory-confirmed NmA meningitis were calculated by dividing the number of meningitis cases in each age-group per 12 months or per month by the population group size. Population estimates were extrapolated from the most recent population census in 2006.

## Results

### Participant Characteristics

In total, 1034 subjects were examined in the study, among whom 1022 consented to have a blood sample and 538 a pharyngeal swab taken. The 12 subjects refusing consent were excluded from all analyses. The mean (+/− SD) age of participants was 18.5 (+/−0.45) years and 53% were female. Eight subjects had a history of meningitis (etiology unknown) and 57 (6%) subjects reported previous meningitis in a family member. Previous meningococcal vaccination was reported by 468 (46%) participants, 69 of whom (7% of total sample) were immunised in 2007–08 and excluded from subsequent serological analyses (but included in carriage estimates). Among the 399 participants reporting vaccination before 2007, most (94%) had received vaccine during campaigns, which dated in Bobo-Dioulasso back to 2002. We included these 399 individuals in the primary analyses, but excluded them in secondary sensitivity analyses. Serology could not be done for 12 sera due to insufficient volumes, yielding 941 participants for seroprevalence estimations.

### SBA and IgG

**Figure 2:**
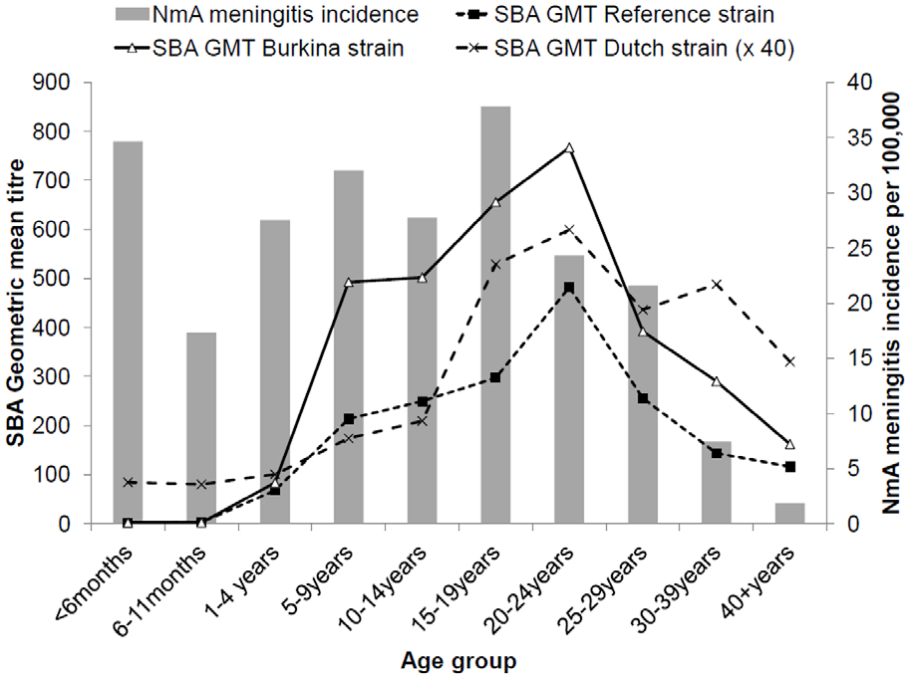
Age-specific serum bactericidal antibody (SBA) geometric mean titers (GMT) against three meningococcal serogroup A assay strains in February–March 2008 *versus* age-specific meningococcal serogroup A meningitis annual incidence during March 2007 through February 2008. General population of Bobo-Dioulasso, Burkina Faso.

**Figure 3:**
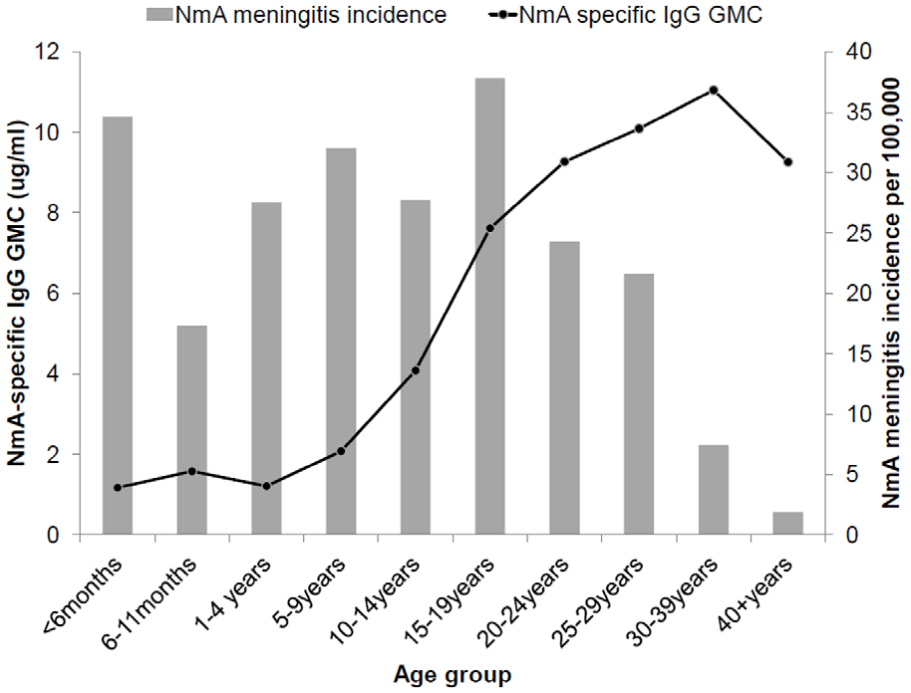
Age-specific serogroup-A specific IgG geometric mean concentrations (GMC) in February–March 2008 versus age-specific meningococcal serogroup A meningitis annual incidence during March 2007 through February 2008. General population of Bobo-Dioulasso, Burkina Faso.

**Figure 4:**
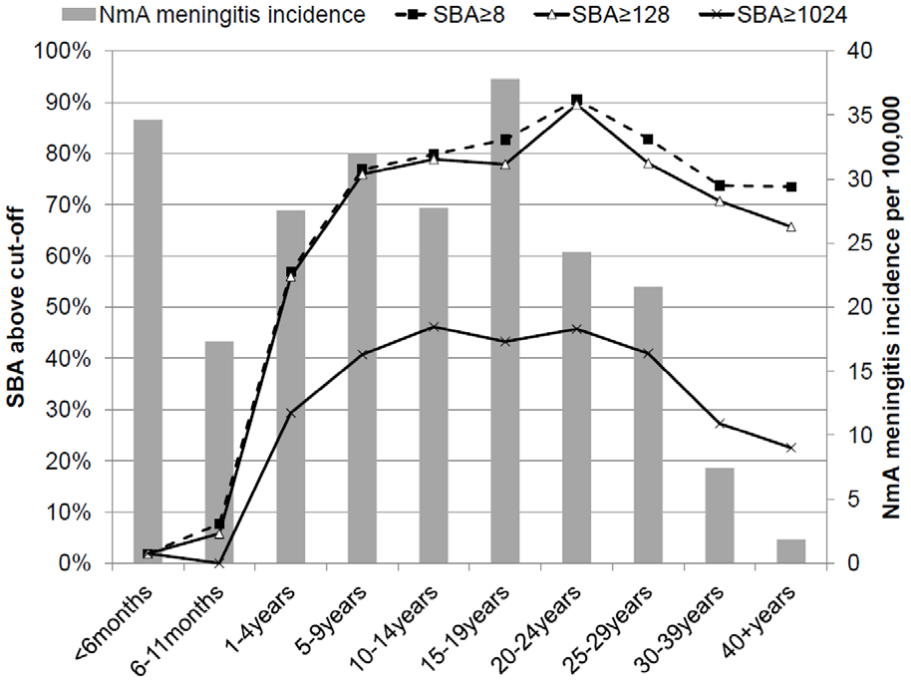
Age-specific prevalence of serum bactericidal antibody (SBA) titers against meningococcal serogroup A equal to or above different cut-offs against the reference strain versus age-specific meningococcal serogroup A meningitis annual incidence during March 2007 through February 2008. General population of Bobo-Dioulasso, Burkina Faso.

SBA GMT against the reference strain was 2 in infants, and increased continuously to a peak of 446 in 20–24 year olds, before declining with age to 103 among 40- to 59-year-old persons (Table 1, Figure 2). The GMC of anti-A IgG increased with age between 5 and 39 years, being above 8 µg/mL, from age 20–24 years and above (Table 1, Figure 3). In children <5 years of age, 27% (95% CI, 21%, 34%) had concentrations ≥2 µg/mL, compared to 93% (92%, 95%) among those aged ≥15 years. Seroprevalence of SBA titers ≥8 or ≥128 showed a similar curve to that for GMT (Figure 4) but with a steep increase to reach maximum levels at age 5 years, decreasing only slightly after age 25 years. The seroprevalence of SBA ≥1024 was lower by a factor of 2 in all age groups.

Seroprevalence estimates restricted to persons without previous vaccination were similar (<5% difference). The frequency distribution of SBA titres against the three different assay strains (Figure 1) showed two peaks, one at 1∶2 and the other between 1∶512 and 1∶2048, starting at 1∶128. We evaluated whether this threshold corresponded to anti-A vaccination before 2007. Controlling for age, individuals reporting meningococcal vaccination prior to 2007 were not more likely to have SBA titers ≥8, ≥128 or ≥1024 against the any of the three strains than unvaccinated individuals (95% CI for odds ratios overlapped 1.0 in all cases).

**Figure 5:**
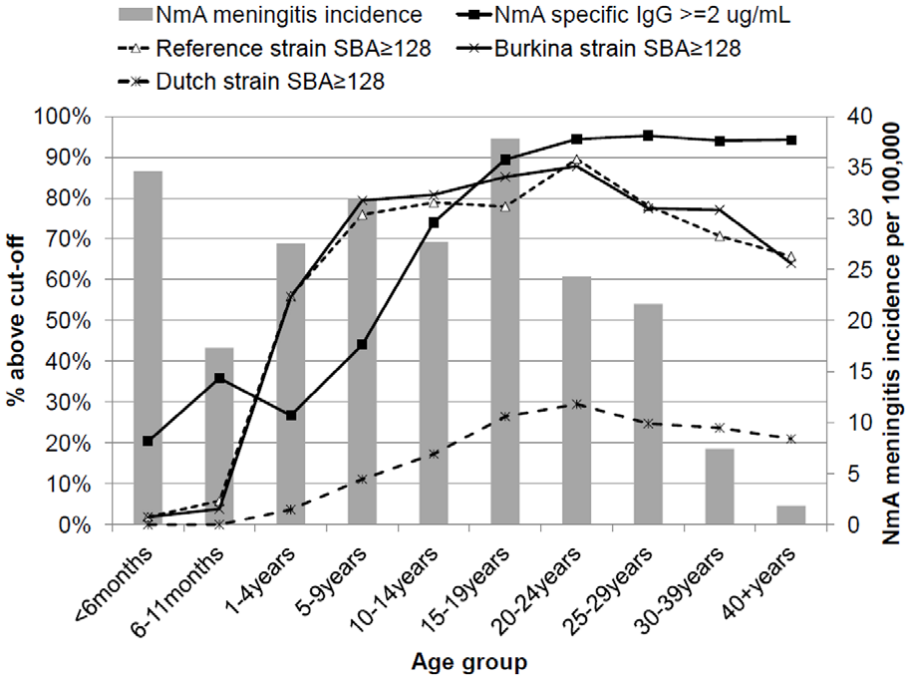
Age-specific prevalence of serum bactericidal antibody (SBA) titers ≥128 against three meningococcal serogroup A assay strains in February–March 2008 and serogroup-A specific IgG ≥2 **ug/mL versus age-specific meningococcal serogroup A meningitis annual incidence during March 2007 through February 2008.** General population of Bobo-Dioulasso, Burkina Faso.

Compared to the reference strain [overall GMT 112 (95%-CI 90, 139)], SBA was significantly higher against the Burkina strain [overall GMT 179 (145, 222)] and significantly lower against the Dutch strain [overall GMT of 6 (5, 7)] (Table 1). Similarly, the proportion of individuals with SBA ≥128 (Figure 5), ≥8 or ≥1024 (data not shown) varied substantially by assay strain. The age-specific patterns using the Burkina or Dutch strain were similar to those for the reference strain with some minor differences (Table 1, Figure 5). The seroprevalence of titers <4 was 2-fold more frequent with the Dutch than other assay strains. The correlation between titres obtained against the reference strain and the Burkina strain was higher (r = 0.79, p<0.001) than that between the reference strain and the Dutch strain (r = 0.37, p<0.001). The correlations between IgG concentration and SBA titres were similar for the reference (r = 0.30) and Burkina strain (r = 0.31) and stronger for the Dutch strain (r = 0.56) (all p<0.001).

Among the 54 infants <6-month-old (median age, 3 months), SBA GMT against any assay strain was ≤2.5 and IgG GMC was 1.2 µg/mL (Table 1). Only one child (1.9%) had elevated titres against the reference and Burkina strains (cut-offs 8-1024), and two children (3.7%) against the Dutch strain.

Other than age, we did not identify any specific risk factors associated with higher antibody titres.

### Carriage

Nine out of 538 participants (1.7%) were identified as meningococcal carriers, including serogroup Y (n = 3), W135 (n = 2), X (n = 1) and non-groupable strains (n = 3) (Table 2). Thirty-four participants (6.3%) were identified as carriers of *Neisseria lactamica*, with an age-specific prevalence declining from 11% in 0- to 14-year-old children to 3% in ≥15-year-old persons.

**Table 2:** Meningococcal isolates in found in nine carriers from a sample of 538 participants.

Carrier	Phenotype	ST	Clonal complex	PFGE ST	PFGE profile
1	Y:14:P1.5,2	4375	23	4375	Y1
2	Y:14:P1.5,2	4375	23	4375	Y4
3	Y:14:P1.5,2	4375	23	4375	Y2
4	NG:4:NST	4899	Unassigned	4899	–
5	NG:14:P1.5,2	6920	Unassigned	6920	–
6	NG:NT:NST	–	–	–	–
7	W135:NT:NST	53	53	23	W1
8	W135:NT:P1.5,2	2881	Unassigned	2881	W2
9	X:15:P1.6	198	198	198	X1

– Indicates not done.

### Disease Incidence

During March 2007 to February 2008, 99 NmA meningitis cases (6 among children <1-year-old) were confirmed by PCR or culture in a population of 594,000. This period incorporated the typical pattern of NmA meningitis with low monthly incidence (mean 0.07/100,000) during May–November 2007 and elevated incidence (mean 3.5/100,000 during) during the dry season months of March–April 2007 and December 2007–February 2008. The annual incidence peaked at around 35/100,000 in infants <6 months of age and 15- to 19-year-olds, and was below 10/100,000 after age 30 years (Figure 2).

No relationship existed between age-specific NmA meningitis incidence estimates and GMT or seroprevalence at any SBA cut-off of the reference or Burkina strain (Figures 2, 3, 4, 5). Among infants, low GMT or SBA seroprevalence were accompanied by high incidence rates and in adults over age 30 years, low incidence was accompanied by high GMT and SBA seroprevalence. However, in individuals aged between 5 and 29 years, both high seroprevalence and high disease incidence were observed. This was similar for IgG ≥2 µg/ml and SBA using the Dutch strain (Figure 5), for which high seroprevalence and incidence overlapped between age 15 and 29 years.

## Discussion

This study describes the patterns of NmA meningitis incidence and natural immunity in the Burkina Faso population before MenAfriVac™ introduction. We found an overall trend from low seroprevalence and high disease incidence in infants towards high seroprevalence and low disease incidence in older adults, with antibody titers peaking at around age 20 years. However, unlike the Goldschneider paradigm (which shows an inverse relationship between age-specific disease risk and immunity [10]) between the ages of 1–20 years, there appeared to be a positive association with rising immunity accompanying increasing meningitis incidence by age. The simplest interpretation of this positive association is that both immunity and disease risk reflect the likelihood of exposure to serogroup A meningococci. These results suggest that that the Goldschneider paradigm [10] cannot directly be transferred to NmA in the meningitis belt and that no straightforward relationship between the currently measured serum antibody and meningitis risk exists in this setting.

Elements that could explain this discrepancy with the Goldschneider curves are that they were produced using strain A16, which is now considered to be too sensitive to complement-mediated killing [20]. Furthermore, environmental, strain and host factors could impact on age-specific antibody functionality and thus render natural immunity less protective. One hypothetical explanation of meningitis seasonality and hyperendemicity in the meningitis belt supposes that during the dry season, colonising meningococci from the nasopharynx directly invade the meninges along the olfactory nerve, facilitated by mucosal damage during the persistent low air humidity [25]. This invasion route would bypass the blood system including serum antibodies. Meningitis incidence during the dry season would therefore be a function of meningococcal transmission (and its age distribution) and mucosal immunity. Such hypothesis needs to be considered critically, as the regular recurrence of epidemic waves strongly suggest, according to general infectious disease dynamics and recent modelling evaluation [26], a major role of acquisition and waning of natural immunity.

Our surveillance data may have some sample size limitations (only 6 cases in children <12 months), but the incidence pattern for the longer time period of March 2007 to December 2009 was comparable. Another limitation may be poor precision of meningococcal vaccination status, which is largely based on recall. It is unlikely that many individuals had received meningococcal vaccination privately, given the low income of the population, or that participants had recently received vaccination during mass campaigns while residing in other districts, as only few campaigns were conducted during 2003 to 2007 in Burkina Faso. The sensitivity analyses excluding any participant reporting previous meningococcal vaccination support this position.

We found high population prevalence of antibody titres which are assumed, in Europe and the US, protective against invasive disease. This is astonishing in the context of concurrent high serogroup A meningitis incidence, the absence of a vaccination programme and lack of identification of NmA colonisation [18]; [27], each of which would lead us to expect low immunity. However, similar high seroprevalence against the reference strain F8238 has been reported from a study in the same population 2 years after A/C polysaccharide vaccination [18] and from unvaccinated 1- to 29-year-old persons in Mali and The Gambia [28]. High seroprevalence could be the result of frequent exposure to NmA itself, or through exposure to cross-reactive antigens [29]–[31] which tend to elicit lower avidity antibodies [32]. While the evidence for very rare NmA colonisation in our study is consistent with similar studies [18]; [27], the substantial incidence of NmA meningitis in this population suggests that NmA does circulate intensively, albeit transiently. Such short exposures may result in antibody development or invasive disease. This interpretation is coherent with two observations in the Burkina Faso population: we previously described a strong cross-sectional association between NmA carriage and anti-A SBA titers during an NmA epidemic [24]; and in a longitudinal carriage and seroprevalence study in Bobo-Dioulasso in the context of hyperendemic NmA meningitis incidence and lack of NmA carriage identification, we found that 50% of healthy persons [4–29 years] without putatively protective immunity against NmA (SBA ≥8 against reference strain) at baseline had developed such immunity after 4 months [18]. Finally, the age distribution of Burkina strain SBA GMT we describe here agrees well with the age distribution of NmA carriage found during the NmA epidemic [24]. Although evidence of high serogroup-specific IgG in the UK [33] and bactericidal antibodies to non-capsular antigens in sera from the USA [32] (where NmA is assumed not to circulate), lend some credence to the argument that at least some of the antibodies measured in our study may be from cross-reactive organisms, to our knowledge, no study has attempted to identify the prevalence of and exposure to such cross-reactive organisms. This alternative explanation of high anti-A seroprevalence is therefore difficult to evaluate.

We found that the results of the SBA assays were sensitive to the choice of the assay strain. SBA results were similar for the reference strain and the Burkina strain, with the higher GMTs for the latter likely reflecting closer antigenic concordance with locally circulating NmA. Overall in our sample, two thirds of individuals had SBA titers ≥128 as measured by the reference strain or the Burkina strain. This contrasts to the much lower levels of immunity as measured using SBA against the Dutch strain (17% ≥128). Differences according to the SBA target strain have been previously described for sera from unvaccinated individuals from several continents [34]. Reference strain F3828 has the immunotype L11, whereas Dutch strain 3125 has immunotype L10, which appears to be less commonly found in carriage. Whilst the NmA strain circulating in Burkina Faso has not been immunotyped, the similarity with the reference strain results suggest that it is L11, or both L10 and L11, as described from the meningitis belt [35]. Poolman *et al* suggested SBA against strain 3125 is a more specific measure of vaccine-induced immunity as it does not capture bactericidal action of antibodies directed against L11, resulting from natural exposure during carriage; while it is equally sensitive to antibody induced by vaccination with serogroup A polysaccharide containing vaccines [34]. The underlying assumption is that immune response to natural lipooligosaccharide immunotype L11 is stronger than that to natural serogroup A capsular polysaccharide. Further experiments to investigate this in a subset of sera are ongoing. Until we have a greater understanding of these differences, it would be prudent to measure individual antibody persistence and population-level immunity after vaccination using both the reference and Dutch strain. Measurement of specific IgG is also sensible, as the only established correlates of protection are based on this [15]; [16], but measured antibodies may be of low or high avidity and thus non-functional, particularly following polysaccharide vaccination [36]. The use of high avidity ELISAs has been deemed of lesser importance following serogroup C conjugate vaccination, which induces primarily high avidity antibodies [37]; [38]. However, no studies to date have been completed on the avidity of serogroup A-specific IgG. The lack of maternal antibodies in children under 6 months of age despite high titers in adults of childbearing age hints that avidity may be low, as it is known that transplacental transfer of high avidity antibodies such as IgG1 is more efficient than that of lower avidity antibodies for example IgG2 [39]; [40].

In conclusion, this study provides estimates of the seroprevalence of naturally-induced anti-NmA antibody in the pre-conjugate vaccine era in the African meningitis belt, according to a variety of antibody measures, which will be helpful to ascertain antibody persistence after MenAfriVac™ introduction. Age-specific seroprevalence of reference strain SBA titres most likely reflects exposure to meningococci and consecutive reactive immunity. We could not define any serological correlate of protection and further work is necessary in this regard, which may include further laboratory development of the current standard methods for measuring antibodies against NmA.
